# Implementing cancer symptom management interventions utilizing patient-reported outcomes: a pre-implementation evaluation of barriers and facilitators

**DOI:** 10.1007/s00520-023-08114-6

**Published:** 2023-11-14

**Authors:** Sarah A. Minteer, Andrea Cheville, Nathan Tesch, Joan M. Griffin, Jessica D. Austin, Sandra Mitchell, Aaron L. Leppin, Jennifer L. Ridgeway

**Affiliations:** 1 Robert D. and Patricia E. Kern Center for the Science of Health Care Delivery, Mayo Clinic, Rochester, MN USA; 2 Department of Physical Medicine and Rehabilitation, Mayo Clinic, Rochester, MN USA; 3 Division of Health Care Delivery Research, Mayo Clinic, Rochester, MN USA; 4 Department of Quantitative Health Sciences, Division of Epidemiology, Mayo Clinic Arizona, Scottsdale, AZ USA; 5 Healthcare Delivery Research Program, National Cancer Institute, Bethesda, MD USA

**Keywords:** Cancer, Symptom management, ePROMS, Pre-implementation evaluation

## Abstract

**Purpose:**

Symptoms can negatively impact quality of life for patients with a history of cancer. Digital, electronic health record (EHR)-integrated approaches to routine symptom monitoring accompanied by evidence-based interventions for symptom management have been explored as a scalable way to improve symptom management, particularly between clinic visits. However, little research has evaluated barriers and facilitators to implementing these approaches in real-world settings, particularly during the pre-implementation phase. Pre-implementation assessment is critical for informing the selection and sequencing of implementation strategies and intervention adaptation. Thus, this study sought to understand pre-implementation perceptions of a remote cancer symptom monitoring and management intervention that uses electronic patient-reported outcome measures for symptom assessment.

**Methods:**

We interviewed 20 clinical and administrative stakeholders from 4 geographic regions within an academic medical center and its affiliated health system during the months prior to initiation of a stepped-wedge, cluster randomized pragmatic trial. Transcripts were coded using the Consolidated Framework for Implementation Research [CFIR] 2.0. Two study team members reviewed coded transcripts to understand how determinants were relevant in the pre-implementation phase of the trial and prepared analytic memos to identify themes.

**Results:**

Findings are summarized in four themes: (1) ability of the intervention to meet patient needs [recipient characteristics], (2) designing with care team needs in mind [innovation design and adaptability], (3) fit of the intervention with existing practice workflows [compatibility], and (4) engaging care teams early [engaging deliverers].

**Conclusion:**

Attention to these aspects when planning intervention protocols can promote intervention compatibility with patients, providers, and practices thereby increasing implementation success.

## Introduction

Patient-reported outcome measures (PROMs) are increasingly used to better understand patients’ health, function, and experiences with healthcare. Research suggests that PROMs may improve symptom detection and treatment in patients with cancer [1], decrease emergency department visits [2] and hospitalizations [3], promote better quality of life and quality-adjusted survival [3], and improve communication between patients and providers [4]. Patient acceptability, willingness to complete oncology PROMs, and patient satisfaction have also been noted [5, 6]. However, clinician uptake and use of PROMs with patients in clinical encounters is variable [7]. Barriers to uptake include time constraints on clinical encounters, potential workflow disruptions, perceptions of limited value added by PROMs, and lack of clinician training to use PROMs [1, 4, 8].

Electronic PROMs (ePROMs) may be a particularly appealing method for identifying cancer symptoms between clinic visits. The COVID-19 pandemic, which decreased the availability of in-person visits, bolstered the prevalence of telehealth but raised questions about how to make care equitable and accessible (e.g., for rural dwelling, impoverished, and older patients and those lacking technological access or knowledge), while ensuring high-quality remote assessment, including with the use of ePROMs [9]. Prior studies in cancer and cancer symptom monitoring have reported feasibility, acceptability, and high survey completion rates for ePROMs, but inconsistencies in how they trigger actions and are used by clinicians remain [10].

Assessments of implementation context during the pre-implementation phase can identify barriers and facilitators to be considered prior to implementing an intervention, which in turn can bolster implementation success and improve uptake among patients and clinicians [6, 11]. Adaptation and fidelity of implementation in the pre-implementation phase can also impact implementation success [12, 13]. A systematic review of reviews found that intervention adaptability, perceived compatibility with existing workflows, and impressions of intervention complexity were important considerations during the pre-implementation design phase, and clinician engagement, training, and resource provision were key facilitators during the planning phase for implementing PROMs in clinical settings [14]. However, only one of the included reviews focused on cancer patients specifically [1]. More recent research has explored barriers and facilitators to implementing PROMs or ePROMs in patients with cancer [15–17], but not in the context of technology facilitated remote symptom management interventions, and only one study was conducted prior to implementation [16].

**Fig. 1:**
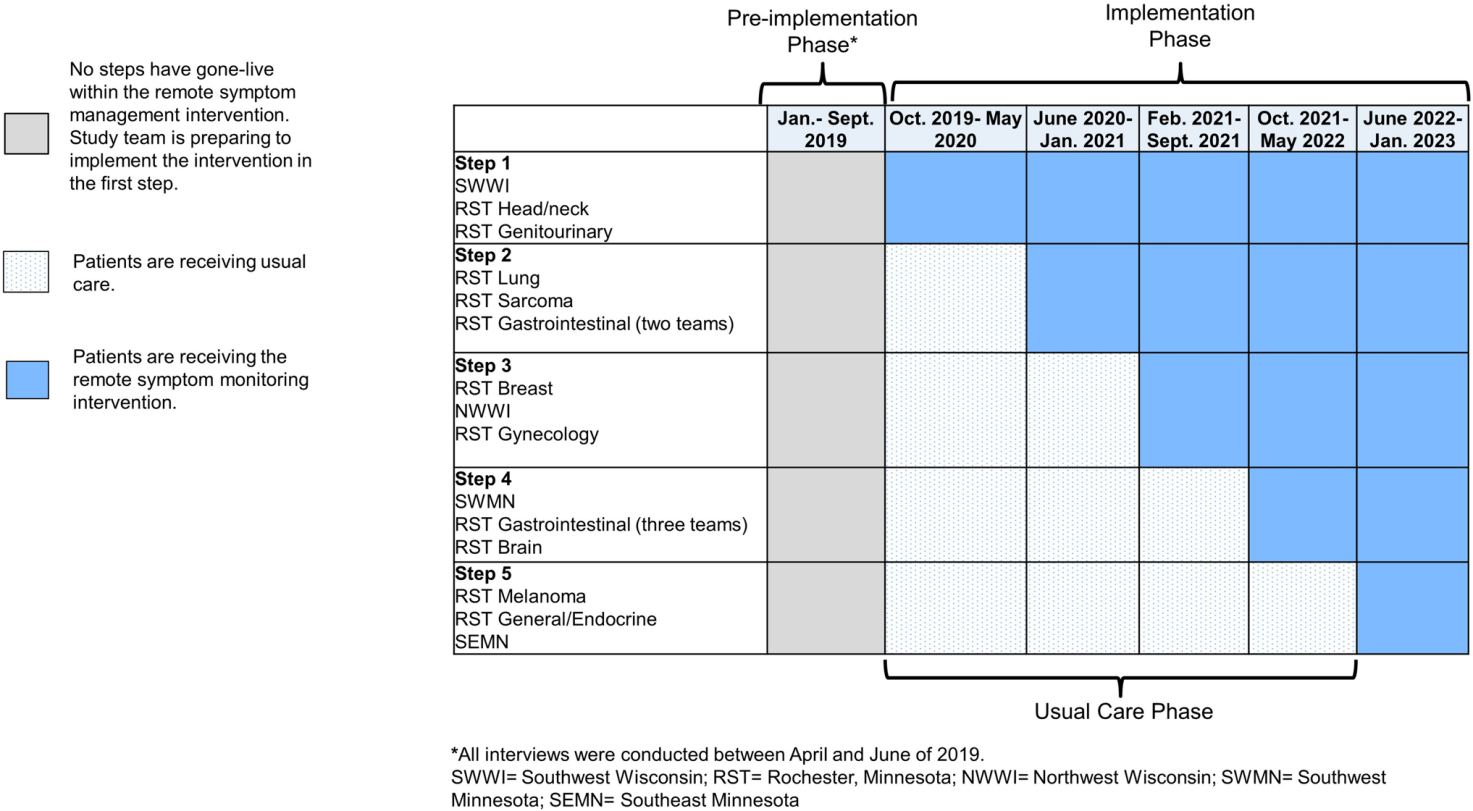
Stepped-wedge trial design showing pre-implementation, usual care, and implementation phases

The present study explored pre-implementation barriers and facilitators to ePROM implementation in the context of initiating a stepped-wedge, cluster randomized pragmatic trial of a remote symptom management intervention, Enhanced EHR (electronic health record)-facilitated Cancer Symptom Control (E2C2) [18] (clinicaltrials.gov Identifier: NCT03892967). In E2C2, patients are assigned ePROMs evaluating sleep, pain, and impairment in physical function, anxiety, depression, and fatigue (low **e**nergy) (making up the SPPADE symptoms) [19] in between oncology visits. The SPADE pentad comprise the most common symptoms experienced by patients in response to disease or treatment side effects [19], and efforts have been undertaken to validate PROM items in these domains along with impairment in physical function to promote standardization [20]. Patients who report moderate or severe symptoms receive self-management materials; telephone support from a registered nurse trained in cancer symptom management (Symptom Care Manager [SCM]) is offered to those with severe symptoms [18]. E2C2 is built upon the evidence-based collaborative care model [21]; SCMs play a central role in patient care while maintaining connections to the larger care team. To identify pre-implementation barriers and facilitators that could inform intervention modifications and implementation strategies, we interviewed administrative and clinical stakeholders during the months before the first step of clinical practices were randomized to the start of the intervention period (see Fig. 1).

## Methods

### Participants and setting

This study took place in a large academic medical center in Rochester, Minnesota (RST), and affiliated community oncology practices in Southwest Minnesota (SWMN), Southeast Minnesota (SEMN), Northwest Wisconsin (NWWI), and Southwest Wisconsin (SWWI) (for additional site characteristics, see Finney Rutten et al., 2020) [18]. As the focus of the pre-implementation assessment was on factors related to implementation of an evidence-based intervention using clinical systems and workflows, these interviews were focused on the perspectives of implementers in the health systems. The study team compiled a list of eligible participants, consisting of those involved with administrative (e.g., operational support, scheduling, and practice decision-making) or clinical functions (e.g., direct patient care) anticipated to be impacted by E2C2. Eligible individuals were invited by email to participate. We scheduled an in-person or telephone interview with those interested. The healthcare system had already instituted PROMs to collect information on patients outside of appointments. Thus, eligible participants may have been somewhat to very familiar with PROMs, depending on their role (e.g., schedulers may have had experience with patients completing PROMs on tablets before visits; physicians may have had experience looking at patients’ responses to PROMs in the EHR). Our goal was to have interviewee representation from each of the sites participating in the trial, so we could identify facilitators and barriers to implementation within different practices and work to address prevalent barriers or strong site-specific barriers. Additional interviews and surveys were planned in each step for the time at which the practices entered the intervention phase and 12 months after it, in order to provide a mechanism for feedback during the trial, including ongoing or emergent facilitators and barriers. Data collection during the trial additionally included eligible patients affiliated with the oncology practice [18]. All procedures were approved by Mayo Clinic’s Institutional Review Board and complied with the Helsinki Declaration. All study participants provided oral consent before participating.

### Data collection and analysis

Interviews were conducted between April and June of 2019. Two implementation scientists (JLR and ALL) conducted interviews using a semi-structured interview guide; questions focused on perceptions of acceptability, appropriateness, and feasibility of the E2C2 intervention, including potential implementation challenges. Participants were asked about existing care delivery approaches for symptom management, clinical workflows, and patient factors (e.g., patient portal use in their local patient population). The interview guide was reviewed by the multidisciplinary team. Interviewers had some familiarity with interviewees prior to the interviews as part of pre-implementation engagement efforts in preparation for implementation of the intervention, but oral consent procedures and consent language described procedures for maintaining confidentiality, and interviewers encouraged both positive and negative views to be shared. Interviews were audio-recorded and transcribed verbatim.

The analytic plan followed methods of directed content analysis, which is a deductive approach guided by existing knowledge about the phenomenon or relevant theory [7, 22]. In this study, we utilized constructs from the Consolidated Framework for Implementation Research (CFIR) 2.0 [23], which is an implementation determinants framework [24] that calls attention to factors that may influence the success of implementation. CFIR is commonly used in implementation science to identify facilitators and barriers to implementation that may arise at any stage of the implementation process; however, fewer studies have used it during the pre-implementation phase, despite this being an important application that can help shape and adapt interventions prior to implementation [25]. Versions of CFIR have been previously applied in deductive approaches to analyzing qualitative data, whereby the framework constructs guided qualitative coding procedures [5, 26], as was the case in our study in which the CFIR 2.0 was used as a codebook to guide line by line deductive coding of transcripts. Two members of the study team (SAM and JLR) met to discuss each coded transcript and resolve discrepancies via discussion, contributing to analytic rigor by dual coding and discussion of each transcript. Final transcripts were uploaded to qualitative analysis software (NVivo, QSR International), and coded text was queried for review. Each investigator wrote an analytic memo [27, 28] with attention to (1) which implementation determinants were most salient in the pre-implementation phase and (2) what the implications were for researchers planning implementation of collaborative care in a busy clinical setting. JLR and SAM met weekly to discuss findings and collaboratively identified a set of core themes.

## Results

We invited 31 stakeholders to participate in an interview and interviewed *N* = 20 stakeholders who agreed to participate; *n* = 12 from Rochester, MN; *n* = 3 from SEMN; *n* = 1 from SWMN;* n* = 1 from SWWI; *n* = 1 who worked in Rochester, MN, and SWMN; and* n* = 2 from the hospital enterprise. Their roles included oncologist (*n* = 8, including one in a leadership role), physical medicine and rehabilitation physician (*n* = 1), nursing leader (*n* = 3), nurse practitioner (*n* = 3), education staff (*n* = 1), IT director (*n* = 1), operations manager (*n* = 1), workflow engineer (*n* = 1), and reception and scheduling supervisor (*n* =1). Interviews lasted 17.65 min on average (standard deviation = 4.88 min, minimum length = 10.27 min, maximum length = 24.52 min).

Pre-implementation findings were summarized in four themes, which mapped onto *five CFIR 2.0 constructs*: (1) ability of the intervention to meet patient needs [*recipient characteristics*]; (2) designing with care team needs in mind [*innovation design* and *adaptability*]; (3) fit of the intervention with existing practice workflows [*compatibility*]; and (4) pre-implementation care team engagement [*engaging deliverers*].

### Qualitative findings

#### Ability of the intervention to meet patient needs [recipient characteristics]

Stakeholders’ perceptions of the intervention were tightly linked to whether they thought this approach to care would benefit patients. ePROM administration was perceived to potentially help manage patients’ symptoms, particularly in between visits. However, stakeholders worried about lack of enhanced processes for addressing patients’ symptoms reported during the usual care phase, particularly if patients reported depressive symptoms prior to their practice starting the intervention phase of the trial. Stakeholders were also concerned about patient care implications if the work of the SCMs overlapped with that of members of the care team (e.g., the clinical team would manage treatment-related nausea, but SCMs would manage sleeplessness and fatigue). They also questioned whether the self-management materials were patient-centered and thought patients might have difficulty using them:

We’re talking a 20-plus page booklet that they’re gonna have to work through to identify what interventions or management tips they should try. They have to first identify their symptom. They have to know which symptoms to try and how that relates to them. (P7; Enterprise Education Staff [develops patient education])

However, the availability of self-management materials in different formats (e.g., articles, videos) was perceived favorably to address different learning styles. Stakeholders noted the importance of understanding each individual patient’s needs, including symptoms, psychosocial needs, level of digital literacy, and broadband access. One stakeholder noted, “Not everyone, especially some of our more rural patients, have computer access on a ready basis.” (P12; Physical Medicine and Rehabilitation Physician)

#### Designing with care team needs in mind [innovation design and adaptability]

Many clinical stakeholders said that they lacked the time and resources to address symptoms during clinic visits. They liked that the intervention would augment the support they provided without impacting their workload directly, but they worried that the intervention could increase the amount and scope of patients’ questions:

I think offering prophylactic and proactive assessment and questioning is a good thing. We just need to have a good sense of what the impact is going to be. Because if you put yourself out there, and you make yourself available more consistently… You might be getting things that maybe require attention that are unrelated to oncology care. (P16; Nurse Practitioner)

Past experiences with external interventionists raised concerns about symptoms being addressed by SCMs external to their oncology team. Stakeholders emphasized the importance of being alerted to more serious symptoms, but not to less serious ones, requiring SCMs to make that determination.

Their biggest concern is that we’re integrating enough, but not too much, with the care teams such that they know what’s going on with the intervention, but not that they’re inundated with extra questions, in-baskets, calls from patients. (P5; Nurse Supervisor)

#### Fit of the intervention with existing practice workflows [compatibility]

Despite their shared health system affiliation, individual practices had different symptom screening and management processes in place. Thus, stakeholders held mixed views on whether the intervention represented an improvement to usual care. For instance, one practice already had robust interdisciplinary workflows and care pathways in place for symptom screening and management:

We have the NCCN distress tool... It meets our QOPI requirements. We have a process here that includes the scheduling staff, the providers, social work, behavioral health, nurse manager, with all of that. That’s really with symptom management, not only physical symptoms, but emotional symptoms. Then we already have occupational therapy embedded. (P20; Oncologist)

A clinical stakeholder from another practice noted that their routine procedures for patient symptom assessment were not accompanied by guideline-based management so this intervention could fill that gap:

The questions when they’re roomed… it does kind of perk my ears up about certain things to pay attention to, but I think without a dedicated intervention to try to help, that becomes less meaningful, and so I think the idea of having both the survey but also the intervention kind of built in is gonna be the most helpful. (P3; Oncologist)

Due to the diversity of practice workflows and patients served, there was tension between stakeholders’ desire to adapt the intervention to fit the needs of their practice and patients and the need for standardization for research purposes. One clinical stakeholder noted:

I think that the input that you get really does have to be implemented. It might not be the same for every site. I know for research it does have to be one size fits all in terms of protocol, and process... (P20; Oncologist)

#### Early and appropriate pre-implementation care team engagement [engaging deliverers]

The remote nature of the intervention was perceived as consistent with innovations in clinical practice (i.e., moving toward technology-enhanced care), but some stakeholders wanted to help adapt and tailor these new approaches. One clinical stakeholder stated:

This is the wave of the future. If we can help shape it and be part of defining how this occurs, all the better for us versus just adopting tools that others have developed. (P10; Oncologist in leadership role)

Some clinical stakeholders expressed concern that much of the E2C2 intervention had been developed without their input: “I think it seems like there was this agenda to get the intervention done without the stakeholders being very involved upfront.” (P16; Nurse Practitioner)

Although it was important to engage stakeholders early, it was also important to share the right amount of information at a given time. In the absence of pertinent information, staff tended to speculate and fill in the gaps:

It’s easy for somebody to hear some details, and then start speculating about things that are going to occur, talk to people about their thoughts that maybe aren’t founded on facts. You start to build this misunderstanding, and sometimes negative views of something that aren’t entirely correct. (P10; Oncologist in leadership role)

Sharing information before things are finalized could also fuel concerns. When information sharing is premature, the implementation team may need to quell concerns that would not have arisen had they waited until the intervention components and implementation strategies had been finalized.

Interviews also highlighted the challenge of randomizing practices to start the intervention at a future timepoint as part of the stepped-wedge trial design from an implementation readiness perspective. One clinical stakeholder stated, “If a cluster is coming, and that clinic happens to be particularly busy, or it’s a particularly busy time of year, it might be a little tougher to do.” (P17; Nurse Practitioner)

### Implications for E2C2 implementation

To address stakeholders’ concerns raised during the interviews, we made several intervention refinements and developed implementation strategies, as shown in Table 1.

**Table 1:** Intervention refinements and implementation strategies developed in response to concerns identifed during pre-implementation

Stakeholder concerns	Intervention refinements and implementation strategies
Ability of the intervention to meet patient needs	
*Need to address severe mental health symptoms patients report during usual care phase*	• Developed procedures for the team to address serious mental health symptoms reported by patients and shared it with care teams
*Some patients lack broadband access or have low digital literacy*	• Investigated and planned for Interactive Voice Response (IVR) telephone option for PROM completion
*Ability of self-management materials to meet patients’ needs*	• Presented clinical stakeholders with evidence supporting the care model • Tailored self-management materials to be symptom specific
Designing with care team needs in mind	
*Increased workload and integration of SCMs with the care team*	• Developed care team materials detailing how SCMs would manage SPPADE symptoms • Requested that SCM and E2C2 study team members periodically join a standing care team meeting to facilitate collaboration, joint problem-solving, care coordination, and role clarification
Fit of the intervention with existing practice workflows	
*ePROMs were duplicative with current symptom assessments*	• Supported some practices’ ASCO Quality Oncology Practice Initiative (QOPI) certification requirements for federal reporting standards by implementing an electronic version of the approved questionnaire and prioritized it to display ahead of other questionnaires patients receive (including our ePROMs assigned for standardization)
Early and appropriate pre-implementation care team engagement	
*Stakeholders were not included in intervention development, so nuances in different practices’ workflows were not adequately addressed*	• Given the duration of usual care for some clusters in the stepped-wedge trial, created a plan to repeat engagement with stakeholders prior to any practices starting the intervention phase of trial • Identified clinical champions and created roles for site-specific implementation facilitators (known as Symptom Sages) to act as a liaison between the care team and our study team and who were knowledgeable in the intervention components, including the EHR clinical decision support elements

## Discussion

User engagement is critical to designing acceptable and feasible interventions, but stakeholders are often asked to implement existing evidence-based practices, as was the case in E2C2. Thus, concerns about E2C2 being appropriate for patients and clinical stakeholders were common. We refined the intervention to address stakeholders’ concerns but could not fully address some. Similar to past research, some participants worried that E2C2 was duplicative of existing symptom assessment or management processes [29, 30]. However, the questionnaire some practices were assigning their patients was similar, but not identical to E2C2 ePROMs, so we still needed to assign our ePROMs to standardize data collection. This demonstrates the tension between needing to preserve intervention fidelity and adapting the intervention to better fit diverse practice workflows.

Some stakeholders’ concerns stemmed from perspectives on what constitutes appropriate cancer care and how the research team was managing the usual care phase of the trial. More specifically, some clinical stakeholders wanted a plan for addressing symptoms reported on ePROMs collected during usual care, above and beyond the standard practice protocols already in place for addressing patients’ ePROM responses. Past research has found providers worry about creating false expectations [16, 30] or issues of liability [31, 32] if symptoms are not or cannot be properly attended to. It may be important to set expectations for the pre-implementation phase, when control data may begin to be collected but practices are still providing usual care, particularly in stepped-wedge trials.

Stakeholders were concerned the intervention could create extra work or require additional time: a common concern about PROMs in cancer care [15, 16, 30, 31, 33]. We designed our intervention to minimize clinical burden by using a combination of patient self-management materials and support from SCMs to address patients’ symptoms. However, stakeholders worried about coordination of symptom management between a patient’s oncology team and SCMs. This represents a unique challenge for collaborative models of care delivery, which include interventionists external to the practice who must keep clinical stakeholders informed without burdening them. Past research has noted the issue of determining the optimal mechanisms and timing of sharing PRO data with clinical stakeholders [34]. Future pre-implementation efforts may consider more in-depth evaluation of what symptoms providers’ wish to be alerted to and how.

Lack of stakeholder engagement during intervention development may have exacerbated existing tensions surrounding the organizational approach to practice improvement. Stakeholders expressed concerns about the lack of collaboration across health system sites and the top-down approach to implementation by the academic medical center. Stakeholders suggested that opinion leaders, who had previously been skeptical but had come to see the value of the intervention, could be particularly effective in helping others overcome concerns and reducing tensions. This approach is consistent with past research noting the importance of clinical champions when implementing PROs [35] or new initiatives [29, 36] in cancer care. We engaged Symptom Sages to champion this initiative to their colleagues, but earlier engagement may have fostered better practice engagement and support.

## Strengths and limitations

This study took the novel approach of characterizing barriers and facilitators to implementation of an ePROM-facilitated remote symptom management intervention for patients with cancer during the pre-implementation phase. Although it is unclear to what extent our findings are generalizable outside our health system, our health system sites did vary in size, rurality, and academic vs. community practice. The majority of those we interviewed worked at our large, academic medical center in Rochester, MN, while fewer interviewees worked at our more rural, community health system sites, which in part reflects differences in staffing and patient volumes. However, it is possible that for smaller health system sites from which we only conducted one interview, perspectives of other staff may have been missed. Nonetheless, these interviews still yielded valuable insights into different clinics’ practice workflows and populations served and identified important barriers and facilitators to implementation. Adding further credibility to our findings is the fact that many of our findings align with past research exploring barriers and facilitators to implementation of PROs. The use of a robust and longstanding implementation determinants framework in analysis may also foster transferability to other settings. Still, it is also possible that our findings suffer from self-selection bias and reflect the views of those who agreed to participate in an interview. However, the fact that interview findings revealed both facilitators and barriers to implementation suggests that interviewees were not limited to those who felt overly positive or negative about the intervention. Lastly, we did not gather patients’ pre-implementation perspectives to help refine the intervention, but their perspectives will be elicited as part of the larger E2C2 trial.

## Conclusion

Our findings demonstrate the importance of pre-implementation assessments when introducing remote symptom monitoring and self-management support interventions in oncology clinics. Some concerns stakeholders had about the intervention were consistent with past research, but others (e.g., wanting a plan for providing enhanced usual care to address symptoms during baseline ePROMs data collection) were unexpected. This latter finding suggests the need to set pre-implementation expectations, especially in the context of pragmatic stepped-wedge trials. The findings presented in this paper may help others in preparing to implement ePROMs and remote symptom management interventions in cancer care and have implications for designing and conducting stepped wedge trials of supportive care interventions in chronic illness settings.

## Data Availability

The data that support the findings of this study are available from the corresponding author upon reasonable request, but restrictions may apply to preserve the rights of participant confidentiality.
